# Evidence-based indications for proton therapy in adults determined using the GRADE approach

**DOI:** 10.1007/s12094-025-04083-w

**Published:** 2026-01-20

**Authors:** Carolina Moltó-Puigmartí, Jordi Giralt, Lucía Alonso García, Clara Pons Duran, Carles Gomà, Sara Pedraza Fernández, Maria-Dolors Estrada Sabadell, Rosa Maria Vivanco Hidalgo

**Affiliations:** 1 AQuAS: Agència de Qualitat i Avaluació Sanitàries de Catalunya (Evaluation Department), Barcelona, Spain; 2 Hospital Universitario Vall d’Hebron (Servicio de Oncología Radioterápica), Universidad Autónoma de Barcelona, Barcelona, Spain; 3 Vall d’Hebron Institut d’Oncologia, Barcelona, Spain; 4 Hospital Clínic de Barcelona, Barcelona, Spain; 5 Hospital Universitario 12 de Octubre, Madrid, Spain; 6 Consorcio de Investigación Biomédica en Red de Epidemiología y Salud Pública (CIBERESP), Madrid, Spain

**Keywords:** Cancer, Proton therapy, Radiotherapy, Hadron therapy, Irradiation

## Abstract

**Abstract:**

**Purpose:**

Proton therapy (PT) offers dosimetric advantages over conventional X-ray–based radiotherapy (XRT), aiming to reduce toxicity and better spare healthy tissues. The Agency for Health Quality and Assessment of Catalonia (AQuAS), commissioned by the Spanish Ministry of Health, conducted a Health Technology Assessment to evaluate the safety and clinical effectiveness of PT for cancer indications not yet approved for PT in Spain. This article summarizes the main findings regarding PT’s safety and clinical performance in adults compared with XRT.

**Methods:**

The assessment was based on a systematic review of primary studies published between 2012 and 2024, following Cochrane methodological standards, PRISMA guidelines, and the GRADE approach. Eligibility criteria were defined using the PICO-DT framework, focusing on adult cancer patients, comparative study designs, and primary outcomes including serious adverse events, mortality, overall survival, and progression-free survival. Risk of bias was evaluated with RoB 2.0 and ROBINS-I depending on study design. Searches covered major biomedical databases.

**Results:**

Of 6958 records screened, 76 were included (five randomized trials and 71 observational studies) across 16 tumour types. Overall, evidence certainty was low or very low, limited by few randomized trials, methodological concerns, and heterogeneity. For some indications, including leptomeningeal metastases, lung cancer, and anal cancer, evidence suggests that PT may be equivalent or superior to XRT, although certainty remains limited.

**Conclusions:**

PT shows variable, cancer-specific results and does not consistently outperform XRT. Some indications appear promising, but substantial evidence gaps persist, emphasizing the need for high-quality comparative studies and systematic clinical data collection.

## Introduction

Proton therapy (PT) is an advanced radiotherapy (RT) modality that uses protons, instead of photons or electrons, to treat cancer. Unlike photon-/X-ray-based radiotherapy (XRT), PT is based on the use of high-energy proton beams that allow the radiation dose to be delivered with high precision [1]. The key advantage of this technology lies in the ability of protons to release most of their energy at a specific point in the tissue, known as the Bragg peak, thus minimizing damage to surrounding healthy tissues. For the same treatment volume, PT significantly reduces the dose to healthy tissue when compared to XRT, even those that use the most advanced technologies, such as intensity-modulated radiation therapy (IMRT) or volumetric modulated arc therapy (VMAT) [2].

The development of PT has been possible thanks to significant advances in particle physics and biomedical engineering. Efforts have also been dedicated to reducing the size of the equipment, enabling its installation in hospitals [3]. Despite its enormous potential, PT still remains a limited resource due to high infrastructure costs and the small number of centers equipped with this technology. Indeed, its use is conditioned by economic factors, infrastructure availability, and the need for more solid clinical evidence. Although the number of PT centers has grown significantly, their availability is still limited [4].

PT has shown advantages over XRT in certain types of cancers, such as pediatric, skull base and spinal cord tumors. However, there is still a lack of solid clinical evidence in other types of tumors to justify its widespread use [5]. This is partly due to its limited accessibility and the difficulty in conducting randomized clinical trials (RCT).

In Spain, PT became part of the common portfolio of services of the National Health System in 2020. The approved indications were very limited: mainly pediatric cancers such as brain tumors or sarcomas close to critical organs and a few adult tumors, including chordomas and chondrosarcomas of the skull base, ocular tumors not candidates for brachytherapy, and paraspinal sarcomas [6]. In 2024, the Agency for Health Quality and Assessment of Catalonia (Agència de Qualitat i Avaluació Sanitàries de Catalunya, AQuAS) published a health technology assessment (HTA) report on the safety, efficacy, clinical effectiveness and efficiency of PT in the treatment of cancer in the adult and pediatric population [7]. The report aimed to update two previous reports [8–10] and systematically review the available evidence on cancer types that had not yet been approved for treatment with PT in Spain. The work was commissioned by the Spanish Ministry of Health, via  the Spanish Network of Agencies for Assessing National Health System Technologies and Performance (Red Española de Agencias de Evaluación de Tecnologías Sanitarias y Prestaciones del Sistema Nacional de Salud, RedETS), and belonged to the 2022 RedETS’ work plan. The objective of this article is to present a summary of the main findings of the report, focusing on the safety, efficacy and clinical effectiveness of PT in the adult population.

## Methods

A systematic review of primary studies was conducted to assess the safety and clinical efficacy/effectiveness of PT compared to XRT, following the methodological standards of the Cochrane Collaboration [11].

### Search strategy

A comprehensive literature search was conducted in January 2024 across several biomedical databases, including Medline (OVID), Embase, the Cochrane Database of Systematic Reviews (CDSR) and Web of Science. The search aimed to identify systematic reviews, RCTs, and comparative observational (CO) studies relevant to the health technology under evaluation. Search strategies combined both controlled vocabulary (e.g., MeSH) and free-text terms, tailored to each database. Boolean operators and filters were applied to limit results to human studies published in the last 10 years, in English, Spanish, or Catalan.

### Study selection criteria

Studies were selected following pre-defined inclusion and exclusion criteria based on the PICO framework (Population, Intervention, Comparator, Outcomes). Eligible studies included pediatric or adult populations with any type of cancer not yet approved for PT treatment in Spain. The intervention was PT for cancer treatment, used either alone or combined with other forms of XRT and/or non-radiation therapies. The comparator was XRT, used either alone or combined with electrons or with other non-radiation therapies. The outcomes were related to safety and efficacy or clinical effectiveness. For safety, both acute and chronic or late adverse events (AEs), whether severe or non-severe, as well as radiation-induced neoplasms, were included. For clinical efficacy/effectiveness, several measures of mortality and survival, disease progression, quality of life, and patient satisfaction or acceptability were considered.

The outcomes considered primary and used for the evidence synthesis and the benefit–risk balance assessment were: acute and chronic or late severe AEs—this is, grade (G) 3 or superior; mortality; overall survival (OS); and progression-free survival (PFS). All other outcomes were considered secondary.

Exclusion criteria encompassed non-comparative studies, editorials, conference abstracts, studies with less than five patients per group, and those published before 2012 or in languages different than Catalan, Spanish, or English.

Unique records were exported to the Covidence^®^ platform [12] for independent, paired screening of title and abstract and full text based on predefined inclusion and exclusion criteria; disagreements were resolved through discussion, without involving additional reviewers.

### Reporting methods and risk of bias assessment

The results of the systematic literature review were reported in accordance with the PRISMA (Preferred Reporting Items for Systematic Reviews and Meta-Analysis) guidelines [13]. Included studies were assessed for quality and risk of bias using standardized tools: RCTs with the Cochrane Risk of Bias tool (RoB 2.0) [14] and CO studies with the risk of bias in non-randomized studies—of interventions tool (ROBINS-I) [15]. Data extraction was conducted using pre-piloted forms, ensuring consistency and completeness.

### Evidence synthesis

The GRADE (Grading of Recommendations Assessment, Development and Evaluation) methodology was applied to present the results of the primary outcomes and to assess the certainty of the evidence [16, 17]. GRADE classifies the certainty of evidence as very low, low, moderate, or high according to the assessment of four domains: risk of bias, inconsistency, indirect evidence and imprecision of the studies providing such evidence.

To facilitate the evidence synthesis, the interpretation of the findings, and the decision-making process, the results were classified into five possible scenarios based on the availability and certainty of the evidence regarding the primary outcomes evaluated, according to GRADE (Table 1).

**Table 1:** Possible scenarios based on the availability and certainty of the evidence

Scenario	Definition
1	There is evidence of sufficient certainty (low or higher) suggesting that PT does not decrease clinical efficacy/effectiveness in terms of OS and PFS, while it maintains or reduces the occurrence of severe acute and chronic toxicity. This scenario would allow recommending or suggesting the use of PT over XRT
2	There is evidence of sufficient certainty (low or higher) suggesting that PT is an equivalent or superior strategy than XRT in at least one primary outcome of both safety and efficacy/effectiveness
3	There is evidence of sufficient certainty (low or higher) suggesting that PT is an equivalent or superior strategy than XRT in at least one primary outcome of either safety or efficacy/effectiveness, but the certainty of the evidence for the other outcomes is very low
4	The evidence tends to indicate that PT could be an equivalent or superior strategy than XRT in terms of safety and efficacy/effectiveness, but the certainty of the evidence is very low
5	It is not possible to assess the balance between the benefits and possible risks of PT compared to XRT due to the absence of data on primary outcomes or because evidence is available only for either safety or efficacy/effectiveness, but not both

## Results

### Results of the bibliographic search and references screening process

**Fig. 1:**
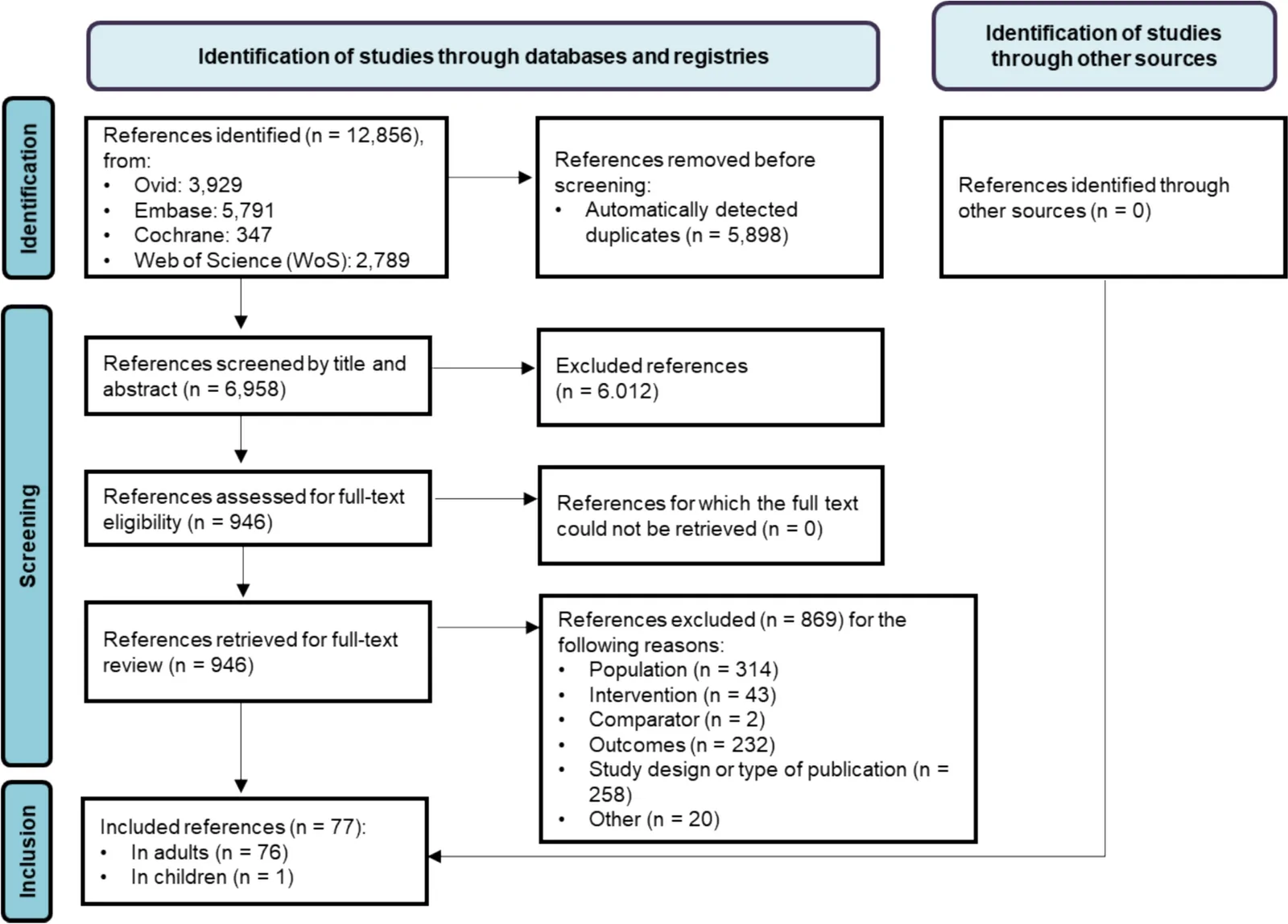
PRISMA flow diagram of the article selection process for safety and clinical efficacy/effectiveness [adapted from [7]]

The literature search yielded a total of 6,958 unique references (after automatically eliminating 5,898 duplicates) which were screened at title and abstract level independently by two reviewers. Of these, 946 were selected for screening of the full text, resulting in 869 references excluded from and 77 included in the evaluation (76 in adults and 1 in children). The study selection workflow is shown in Fig. 1.

### Results by cancer type

A total of 76 studies performed on adults and meeting the selection criteria were identified. Of these, 5 were RCTs [18–22] and 71 were CO studies [23–94]. Fifteen clinical indications or tumor types were identified and grouped into six categories based on anatomical location or body system: head and neck, central nervous system (CNS), thorax, digestive system, male genital system, breast and female reproductive system. Table 2 presents the total number of studies included in the evaluation by indication and study design; Table 3 summarizes the main characteristics of the studies; finally, Table 4 shows the tumor distribution across the different scenarios of evidence according to the definitions of Table 1.

**Table 2:** Number of included studies, classified by indication and study design

Anatomical location	Study design	
	RCT	CO
Head and neck		
1. Oral cavity and pharyngeal cancer		9
2. Nasopharynx		5
Central nervous system		
3. Glioma and glioblastoma	1	4
4. Medulloblastoma		1
5. Acoustic neuroma		1
6. Leptomeningeal metastasis	1	
Thorax		
7. Lung cancer	1	14
Digestive system		
8. Esophageal cancer	1	9
9. Pancreatic cancer		1
10. Hepatocellular carcinoma		5
11. Anal cancer		1
Male genital system		
12. Prostate cancer	1	13
13. Testicular cancer		1
Breast and female reproductive system		
14. Breast cancer		5
15. Uterine cancer		2
Total studies included	5	71

*RCT* randomized controlled trial, *CO* comparative observational study

**Table 3:** Main characteristics of the included studies

Primary site first author, year	Study design intervention and comparator	Total nr of participants (PT and n XT)	Type of tumor and stage	Treatment intent	Corresponding tables in AQuAS HTA report [7]
Head and neck					
1. Oral cavity and pharyngeal cancer					
Yoon, 2021	CO study with between-group matching IMRT + IMPT and IMRT with concurrent systemic therapy	*N* = 72 (36 and 36)	Oropharyngeal cancer III–IV	Definitive	Table 57 (study characteristics) Table 10 (study results)
Blanchard, 2016	CO study with between G matching IMPT and IMRT	*N* = 150 (50 and 100)	Oropharyngeal cancer T1–T2/N2–N3	Curative	
Bagley, 2020	CO study without GM Passive-scattering PT or IMPT and IMRT or SBRT	*N* = 69 (14 PT and 40 IMRT + 15 SBRT)	Recurrent oropharyngeal cancer or second primary tumor, stage NR	Curative	
Manzar, 2020	CO study without GM IMPT and VMAT	*N* = 305 (46 and 259)	Oropharyngeal cancer I–IVC	Definitive or adjuvant	
Romesser, 2016	CO study without GM PT and IMRT	*N* = 41 (18 and 23)	Salivary gland cancer or metastasis	Definitive or adjuvant	
Youssef, 2022	CO study without GM IMPT and IMRT	*N* = 292 (58 and 234)	Oropharyngeal cancer T1–T4/N0–N3	Curative	
Sharma, 2018	CO prospective study without group matching PT and VMAT	*N* = 64 (31 and 33)	Oropharyngeal squamous cell carcinoma I–IVA, T1–T3–Tis, N1–N3	Adjuvant	
Sio, 2016	CO study without GM IMPT and IMRT	*N* = 81 (35 and 46)	Oropharyngeal cancer T1–T4, N0–N3, TNM I–IVA–B	Definitive	
Zhang, 2017	CO study without GM IMPT and IMRT	*N* = 584 (50 and 534)	Oropharyngeal cancer T1–T4/N0–N3	Definitive	
2. Nasopharynx					
Chou, 2021	CO study with between–group matching IMPT and VMAT	*N* = 160 (80 and 80)	Nasopharyngeal cancer T1–T4/N0–N3; I–IV	NR	Table 58 (study characteristics) Table 11 (study results)
Wu, 2022	CO study with between–group matching IMPT and VMAT	*N* = 107 (53 and 54)	Nasopharyngeal carcinoma I–IV, T1–T4, N0–N3	NR	
Li, 2021	CO study without GM IMPT and IMRT	*N* = 77 (28 and 49)	Nasopharyngeal Carcinoma T1–T4/N0–N3; I–IVA	Curative	
Alterio, 2020	CO study without GM IMRT followed by PT boost and IMRT	*N* = 44 (27 and 17)	Locally advanced nasopharyngeal carcinoma T3–T4/N0–N3/M0; III–IVA	Curative, as a boost	
McDonald, 2016	CO study without GM PT and IMRT	*N* = 40 (14 and 26)	Nasopharynx, nasal or paranasal sinuses cancer T1–T4, N0–N2	Definitive or adjuvant	
Central nervous system					
3. Glioma and glioblastoma					
Brown, 2021	RCT IMPT or passive scattered PT and IMRT	*N* = 67 (26 and 41)	Glioblastoma or o gliosarcoma IV	Curative	Table 59 (study characteristics) Table 12 (study results)
Adeberg, 2017	CO study with between-group matching Proton boost following chemoradiation with photons and XT	*N* = 132 (66 and 66)	High-grade glioma	Curative	
Matsuda, 2023	CO study with between-group matching PT and XT	*N* = 52 (26 and 26)	Glioblastoma, stage NR	Adjuvant	
Jhaveri, 2018	CO study with between-group matching PT and XT (IMRT, 3D-CRT)	*N* = 49,575 (170 and 49,405)	Gliomas I–IV	Curative	
Ritterbusch, 2021	CO study without GM PT (uniform or pencil-beam scanning) and XT	*N* = 100 (57 and 43)	Oligodendroglioma or astrocytoma II–III	NR	
4. Medulloblastoma					
Brown, 2013	CO study without GM Craniospinal PT and focal XT	*N* = 40 (19 and 21)	Medulloblastoma, stage NR	Adjuvant	Table 60 (study characteristics) Table 13 (results)
5. Acoustic neuroma/schwannoma					
Kuchler, 2022	CO study without GM Fractionated PT and radiosurgery, stereotactic hypofractionated RT or XT	*N* = 261 (25 and 236)	Acoustic neuroma or vestibular schwannomas T1–T4b	NR	Table 61 (study characteristics) Table 14 (study results)
6. Leptomeningeal metastasis					
Yang, 2022	RCT Hypofractionated PT and XT	*N* = 63 (42 and 21)	Leptomeningeal metastasis of NSCLC or breast cancer primary	Palliative	Table 62 (study characteristics) Table 15 (study results)
Thorax					
7. Lung cancer					
Liao, 2018	RCT Passive scattering PT and IMRT	*N* = 149 (57 and 92)	NSCLC IIA–IV	Definitive	Table 63 (study characteristics) Table 16 (study results)
Suh, 2022	CO study with between-group matching PT and XT	*N* = 229 (112 and 117)	NSCLC (mainly squamous cell and adenocarcinoma) T1a–T2a	Definitive	
Wang, 2016	CO study with between-group matching PBT (passive scattered) and 3D-CRT or IMRT	*N* = (26 and 56)	Primary or recurrent, locally advanced and unresectable NSCLC I–III	NR	
Higgins, 2017	CO study with between-group matching PT and XT (external beam not specified photon RT)	*N* = 616 (308 and 308)	NSCLC (adenocarcinoma, squamous cell carcinoma, other) 0–IV	NR	
Kim, 2021	CO study with between-group matching Pencil beam scanning PT and IMRT	*N* = 75 (25 and 50)	NSCLC T1–T4/N2–N3;IIIA–IIIC	Definitive	
Boyce Fappiano, 2021	CO study without GM PT and IMRT	*N* = 136 (61 and 75)	NSCLC T1–T4/N0–N2/M0–M1; II–IV	Adjuvant	
Kim, 2019	CO study with between–group matching PT and XT	*N* = 30 (8 and 22)	NSCLC IA–IIB	Definitive	
Remick, 2017	CO study without GM Post-operative PT or IMRT	*N* = 61 (27 and 34)	NSCLC T1–T4/N0–N3; IA- > = IIIA	Adjuvant	
Yang, 2022	CO study without GM Re-irradiation with PT or XT	*N* = 63 (22 and 41)	Locoregional recurrent NSCLC I–III	Salvage	
Yu, 2019	CO study without GM IMPT and IMRT	*N* = 79 (33 and 46)	Locally advanced NSCLC, I–IV	Curative	
Yu, 2022	CO study without GM IMPT and IMRT	*N* = 163 (35 and 128)	Biospy-proven primary or recurrent NSCLC	Definitive	
Zou, 2020	CO study without GM PT (beam or uniform scanning) and IMRT	*N* = 64 (34 and 30)	NSCLC, small-cell, T1–T4/N0–N3;IIIA–IIIC	Curative	
Cortiula, 2024	CO study without GM IMPT and IMRT(both with concurrent chemo-RT)	*N* = 271 (71 and 200)	NSCLC (squamous and non- squamous) IIIA–IIIC	Curative	
Seo, 2023	CO study without GM PT and photon XT	*N* = 262 (20 and 242)	NSCLC I–III	Definitive	
Palma, 2020	CO study without GM Passive scattered PT and IMRT (both with CT–RT)	*N* = 166 (63 and 103)	Inoperable NSCLC II–IIIB	Definitive	
Digestive system					
8. Esophageal cancer					
Lin, 2020	RCT PT (scattered or beam) and IMRT	*N* = 107 (46 and 61)	Locally advanced esophageal cancer I–III	Neoadjuvant or definitive	Table 64 (study characteristics) Table 17 (study results)
Fang, 2018	CO study with between-group matching PBT and IMRT	*N* = 220 (110 and 110)	Esophageal cancer (adenocarcinoma and squamous cell carcinoma) I–IVA	Definitive	
Shiraishi, 2018	CO study with between-group matching PBT and IMRT	*N* = 272 (136 and 136)	Esophageal cancer I–IVA	Neoadjuvant	
Bhangoo, 2020	CO study without GM IMPT and IMRT	*N* = 64 (32 and 32)	Esophageal adenocarcinoma or squamous cell T1–3, N0–3	Neoadjuvant or definitive	
Choi, 2022	CO study without GM PT and XT	*N* = 31 (15 and 16)	Esophageal squamous cell carcinoma T1–4, N1–3	Neoadjuvant	
DeCesaris, 2020	CO study without GM PBT (pencil-beam) and XT	*N* = 54 (18 and 36)	Adenocarcinoma of the esophagus/gastroesophageal junction T2–4, N0–2	Neoadjuvant	
Makishima, 2015	CO study without GM PT and XT	*N* = 44 (25 and 19)	Esophageal cancer 0–IIIC	Definitive	
Suh, 2021	CO study without GM PT and XT	*N* = 77 (48 and 29)	Esophageal cancer T1–T3	Definitive	
Xi, 2017	CO study without GM PT and IMRT	*N* = 343 (132 and 211)	Thoracic esophageal carcinomaT1–4, N0	Definitive	
Garant, 2019	CO study without GM PT (pencil beam) and 3D-CRT or IMRT	*N* = 125 (62 and 63)	Esophageal or gastroesophageal junction carcinoma I–III and other	Neoadjuvant or definitive	
9. Pancreatic cancer					
Maemura, 2017	CO study without GM PT and hyper-fractionated acceleration RT	*N* = 25 (10 and 15)	Unresectable locally advanced pancreatic cancer, stage NR	NR	Table 65 (study characteristics) Table 18 (results)
10. Hepatocellular carcinoma					
Cheng, 2020	CO study with between-group matching PT and XT	*N* = 110 (55 and 55)	Primary hepatocellular carcinoma I–IVA	Curative	Table 66 (study characteristics) Table 19 (study results)
Hasan, 2019	CO study with between-group matching PT and SBRT	*N* = 112 (56 and 56)	Early stage unresectable hepatocellular carcinoma T1–2/N0	Curative	
Sanford, 2019	CO study without GM PT and IMRT or VMAT	*N* = 133 (49 and 84)	Nonmetastatic unresectable hepatocellular carcinoma	Ablative	
De, 2021	CO study without GM PT and IMRT or VMAT	*N* = 143 (40 and 103)	Unresectable hepatocellular carcinoma I–IV	Definitive	
Hsieh, 2024	CO study without GM PT (passive scatter) and SBRT or IMRT	*N* = 159 (105 and 54)	Inoperable hepatocellular carcinoma, stage NR	Definitive	
11. Anal cancer					
Mohiuddin, 2021	CO study without GM IMPT and IMRT	*N* = 208 (58 and 150)	Carcinoma of the anal canal I–IV, T0–T4, N0–N1c, M0–M1	Definitive	Table 67 (study characteristics) Table 20 (results)
Male genital system					
12. Prostate cancer					
Khmelevsky, 2018	RCT XT + PT boost and XT	*N* = 289 (116 and 173)	Locally advanced prostate cancer T1–4N0, T2–3N1M0	NR	Table 68 (study characteristics) Table 21 (study results)
Barsky, 2021	CO study with between-group matching Postoperative PT and IMRT	*N* = 260 (65 and 195)	Prostate cancer ≤ T3	Adjuvant or salvage	
Dutz, 2019	CO study with between-group matching PT and IMRT	*N* = 58 (29 and 29)	Localized or locally advanced prostate cancer, stage NR	Definitive	
Fang, 2015	CO study with between-group matching PT and IMRT	*N* = 188 (94 and 94)	Localized prostate adenocarcinoma (no extraprostatic disease or pelvic node involvement)	Definitive	
Liu, 2021	CO study with between-group matching PT and IMRT or 3D-CRT	*N* = 3.720 (1.860 and 1.860)	Localized prostate cancer T1–T3	Definitive	
Pan, 2018	CO study with between-group matching PT and IMRT	*N* = 4.158 (693 and 3.465)	Prostate cancer, stage NR	NR	
Santos, 2019	CO study with between-group matching PT and IMRT	*N* = 267 (70 and 197)	Prostate adenocarcinoma T2–3, N0–1	Adjuvant or salvage	
Sheets, 2012	CO study with between-group matching PT and IMRT	*N* = 1.368 (684 and 684)	Primary localized prostate cancer T1–T4	Definitive	
Yu, 2013	CO study with between-group matching PT and IMRT	*N* = 1.263 (421 and 842)	Early stage prostate cancer, stage NR	Definitive	
Bai, 2020	CO study without GM PT and IMRT	*N* = 262 (105 and 157)	Clinically localized prostate cancer T1–T2	Definitive	
Gray, 2013	CO study without GM PT and IMRT	*N* = 248 (95 and 153)	Localized prostate cancer T1–T3	NR	
Hoppe, 2014	CO study without GM PT (passive scatter) and IMRT	*N* = 1.447 (1.243 and 204)	Localized prostate cancer T1–T3	NR	
Lukez, 2023	CO study without GM moderate hypofractionated PT and IMRT	*N* = 772 (485 and 287)	Localized prostate cancer T1c–T2c	Definitive	
Vapiwala, 2021	CO study without GM moderate hypofractionated PT and IMRT	*N* = 1.850 (568 and 1.282)	Early stage prostate adenocarcinoma T1c–2	Definitive	
13. Testicular cancer					
Pasalic, 2020	CO study without GM PT and 3D-CRT	*N* = 55 (11 and 44)	Testicular seminoma IA–IIC, T1–T3, N0–N3	Adjuvant	Table 69 (study characteristics) Table 22 (study results)
Breast and female reproductive system					
14. Breast cancer					
Chowdhary, 2019	CO study without GM PT and XT (with or without electron boost)	*N* = 724.492 (871 and 723.621)	Non metastatic breast cancer 0–III, pT0–pT4, pN0–pN3	Adjuvant	Table 70 (study characteristics) Table 23 (study results)
DeCesaris, 2019	CO study without GM PT (pencil beam) and XT	*N* = 86 (39 and 47)	Primary invasive breast cancer IA–IIIC	Adjuvant	
Galland-Girodet, 2014	CO study without GM PBT and 3D-CRT	*N* = 98 (19 and 79)	Invasive breast carcinoma pT1N0M0	Adjuvant	
Sayan, 2023	CO study without GM PT (not specified) and XT	*N* = 37 (11 and 26)	Breast cancer T1–T4 and N0–N3	Adjuvant	
Teichman, 2018	CO study without GM Hypofractionated partial PT and whole breast XT	*N* = 129 (72 and 57)	Early stage breast cancer 0–II	Adjuvant	
15. Uterine cancer					
Anderson, 2022	CO study without GM PT (not specified) and IMRT	*N* = 47 (2 and 45)	Endometrial cancer I–IV	Adjuvant	Table 71 (study characteristics) Table 24 (study results)
Xu, 2018	CO study without GM PT (pencil beam) and IMRT	*N* = 25 (7 and 18)	Locally advanced endometrial cancer, stage NR	NR	

*3D-CRT* 3D-conformal radiotherapy, *CO* comparative observational study, *GM* group matching, *IMPT* intensity-modulated proton therapy, *XT* photon radiotherapy, *IMRT* intensity-modulated radiation therapy, *NR* Non reported, *NSCLC* non-small-cell lung cancer, *PT* proton therapy, *RCT* randomized controlled trial, *SBRT* stereotactic body radiation therapy, *VMAT* volumetric modulated arc therapy.

**Table 4:** Tumor distribution across the different scenarios of evidence for proton therapy (PT) vs X-ray-based radiotherapy (XRT)

Scenario	Type of tumor
1. Sufficient evidence—PT equal or better than XRT (all primary outcomes of efficacy and toxicity)	None
2. Sufficient evidence—PT ≥ XRT (at least 1 primary outcome of efficacy and safety)	Cerebral leptomeningeal metastasis, lung, anal
3. Sufficient evidence—PT ≥ XRT (at least 1 primary outcome of efficacy or safety); very low certainty in the other primary outcomes	Gliomas, esophageal, prostate
4. Very low certainty—PT ≥ XRT	Oral cavity/pharynx, sinonasal, pancreas, liver, breast
5. Insufficient evidence—not possible to assess the benefit–risk balance (only outcomes of efficacy or only of safety)	Testicular, medulloblastoma, acoustic neuroma or vestibular schwannoma, uterine

### Head and neck

#### Oral cavity and oropharyngeal cancer

Nine studies were included, 2 with a CO design with between-group matching [24, 27] and 7 with an unmatched CO design [23, 25, 26, 28–30, 90]. RT was used with a radical or adjuvant intent.

Few or no differences between PT and XRT were found regarding most of the severe acute AE analyzed [23, 26–28] and severe chronic AE [23, 28, 30]. One CO study [27] found evidence in favor of PT for acute G3/4 mucositis. In terms of clinical effectiveness, again few or no differences between PT and XRT were found [24–28]. The certainty of the evidence was very low for all of the studied outcomes. We classified this indication in scenario nr. 4 (Table 1).

#### Nasopharynx

Five studies were included, 2 studies with a CO design with between-group matching [33, 36] and 3 with an unmatched CO design [32, 34, 35]. Three of the five studies had radical or adjuvant intent; in the other two, the intent was not reported [33, 36].

For most of the severe acute AE, there were few or no differences between PT and XRT. However, evidence on G3 mucositis was in favor of PT and that on G3 dermatitis in favor of XRT. Regarding severe chronic AE, few or no differences were found between PT and XRT. Regarding clinical effectiveness, in general the evidence revealed few or no differences between PT and XRT. In some cases, evidence favored PT in mortality or OS outcomes. Importantly, no evidence was found against the use of PT. The certainty of the evidence was very low for all outcomes of safety and clinical effectiveness. We classified this indication in scenario nr. 4 (Table 1).

### Central nervous system

#### Gliomas and glioblastomas

Five studies were included, 1 RCT [18], 3 studies with a CO design with between-group matching [37–39] and 1 unmatched CO study [40]. The studies used external RT with radical or adjuvant intent [18, 37–39], or not reported [40].

Regarding acute G3 AEs, in 1 RCT [18] and in 2 CO studies [37, 39], few or no differences were found between PT and XRT. In 1 CO study, few or no differences were found in the frequency of late G3 AEs [39]. In terms of clinical effectiveness, 1 RCT found little or no difference with respect to median OS and PFS time between the PT and XRT groups [18]. Based on the CO studies, PT led to longer OS than XRT in 2 studies [38, 39], no difference was observed in 1 study [40], and PT was associated with shorter OS in 1 study [37]. The evidence from OS studies pointed to equal or better PFS outcomes with PT than with XRT [37, 39]. The certainty of the evidence was low in one domain but very low in the rest. In addition, one study pointed to a possible worse 1-year OS in the group treated with PT. Overall, we classified this indication in scenario nr. 3 (Table 1).

#### Medulloblastoma

One study with a CO design without group matching was included [41]. The study used external RT with adjuvant intent.

No evidence was found for acute or severe chronic AEs of PT compared to XRT. In terms of clinical effectiveness the study reported few or no differences between techniques. The certainty of the evidence was very low for all outcomes of clinical effectiveness. We classified this indication in scenario nr. 5 (Table 1).

#### Acoustic neuroma or vestibular schwannoma

One study with a CO design without group matching was included [42]. The treatment intent was not reported.

No evidence was found on severe acute AEs of PT compared to XRT, while few or no differences were reported regarding severe chronic AEs (hearing impairment according to Gardner–Robertson, classes III–IV, at 60 months). In terms of clinical effectiveness, no differences were found in either PFS or mortality. The certainty of the evidence was very low for all outcomes evaluated. We classified this indication in scenario nr. 5 (Table 1).

#### Leptomeningeal metastasis

One RCT was included [19]. The study used external RT with palliative intent. No differences were found in the frequency of on-treatment G3 and G4 adverse events and G3 and G4 lymphopenia, while no evidence was identified on chronic toxicity. In terms of clinical effectiveness, PT resulted in longer median survival time and CNS PFS time. It is important to note that the treatment volumes differed between the two arms (cranioespinal for PT; local for XRT), which could have influenced disease control. The certainty of the evidence was low for at least one outcome of safety and clinical effectiveness. We classified this indication in scenario nr. 2 (Table 1).

### Lung cancer

Fifteen studies were included, 1 RCT [20], 4 studies with a CO design with between-group matching [69, 71, 80, 83] and 10 with an unmatched CO design [65, 70, 72, 76, 85, 87–89, 91, 94]. Ten studies had radical intent [20, 70–72, 80, 87–89, 91, 94], 3 salvage [85] or adjuvant intent [65, 76] and 2 did not report the intent of the treatment [69, 83].

Most studies found no differences between PT and XRT in terms of severe acute adverse AEs, although evidence was also found both in favor of and against PT. Most studies, including one RCT, reported few or no differences between PT and XRT in severe chronic AEs, although one study [88] found evidence in favor of PT. Regarding efficacy/clinical effectiveness, most studies—including the RCT—reported few or no differences between PT and XRT in OS and PFS, although some evidence in favor of PT was identified [65]. The certainty of the evidence was moderate for OS time, low for G3 radiation pneumonitis, and very low for the remaining outcomes assessed. We classified this indication in scenario nr. 2 (Table 1).

### Digestive system

#### Esophageal cancer

Ten studies were included, 1 RCT [21], 2 studies with a matched CO design [49, 51] and 7 with an unmatched CO design [43–48, 50]. The studies evaluated RT with radical or neoadjuvant intent.

The majority of the studies found few or no differences between PT and XRT with respect to severe acute or chronic AEs. In terms of efficacy/clinical effectiveness, most studies found few or no differences between PT and XRT regarding OS and PFS. Evidence was also found in OC studies in favor of PT for both types of outcomes. The certainty of the evidence was low for the outcome of G3 + acute AEs (treatment-related and non-treatment-related) and very low for the remaining outcomes. Overall, we classified this indication in scenario nr. 3 (Table 1).

#### Pancreatic cancer

One study with a CO design without group matching was included [52]. The study did not report the treatment intent.

The study found no differences between PT and XRT in terms of severe acute AEs and clinical effectiveness outcomes, while it did not report results on severe chronic AEs. The certainty of the evidence was very low for all outcomes. Overall, we classified this indication in scenario nr. 4 (Table 1).

#### Hepatocellular carcinoma

Five OC studies were included—two with group matching design [53, 54] and three without matching [55, 56, 92]. All studies assessed external RT with radical [53, 54, 56, 92] or ablative intent [55].

Most of the identified studies reported results in favor of PT regarding severe acute AE, while no study reported results on severe chronic AEs. In terms of clinical effectiveness, all identified studies reported results in favor of PT for both median OS time and OS at 1, 2, and 3 years. Evidence was also found in favor of PT with regard to the median PFS time and PFS up to 3 years. The certainty of the evidence was very low for all outcomes, both safety- and effectiveness-related. Overall, we classified this indication in scenario nr. 4 (Table 1).

#### Anal cancer (anal squamous cell carcinoma)

One study with a CO design was included [63]. External RT was used with radical intent.

The study found few or no differences between PT and XRT in severe acute and chronic AEs. In terms of clinical effectiveness, no evidence was identified regarding OS, while few or no differences were found between PT and XRT in PFS. The certainty of the evidence was low. We classified this indication in scenario nr. 2 (Table 1).

### Male genital system

#### Prostate cancer

Fourteen studies were included: 1 RCT [22], 8 CO with group matching [58, 59, 62, 74, 77, 79, 86, 95], and 5 CO without group matching [57, 60, 61, 82, 93]. Of the 14 studies, 10 used external RT for radical, adjuvant, or salvage intent [57–59, 62, 77, 79, 82, 86, 93, 95], while 4 studies did not specify the treatment intent [22, 60, 61, 74].

The majority of the identified studies found few or no differences between PT and XRT with respect to severe acute and chronic AEs. In terms of efficacy/clinical effectiveness, most of the studies, both OC and RCTs, found few or no differences between techniques regarding OS and mortality, although evidence was also found in favor of PT for OS. No evidence was found regarding PFS. The certainty of the evidence was moderate for G3/4 genitourinary and gastrointestinal acute AEs, low for G3/4 chronic or late gastrointestinal AEs, and very low for the remaining outcomes studied. Overall, we classified this indication in scenario nr. 3 (Table 1).

#### Testicular cancer

One unmatched cohort study [75] was included, involving patients with testicular seminoma treated either with PT (*n* = 11) or 3DRT (*n* = 44). External RT was used with adjuvant intent.

No evidence was found regarding any safety or clinical effectiveness primary outcomes. We classified this indication in scenario nr. 5 (Table 1).

### Breast and female reproductive system

#### Breast cancer

Five unmatched cohort studies were included [66–68, 78, 81]. In most of the studies, the objective was to compare acute toxicity data. In general, cohort sizes were small. External RT was used with adjuvant intent.

The majority of the studies found few or no differences between PT and XRT regarding severe acute AEs. Studies where no events occurred were also identified. None of the identified studies reported results on severe chronic AEs. In terms of clinical effectiveness, evidence in favor of PT for 5-year OS was reported in one study, and no studies reported results on PFS. The certainty of the evidence was very low for all outcomes, both safety- and effectiveness-related. Overall, we classified this indication in scenario nr. 4 (Table 1).

#### Uterine cancer

Two unmatched cohort studies were included [64, 84], aiming to assess differences in acute toxicity following pelvic irradiation [64] or pelvic and para-aortic irradiation [84]. External RT was used with adjuvant intent [64] or the intent was not reported [84].

No differences were found in the frequency of total G3/4 acute AEs between groups. No evidence was found regarding progression or mortality primary outcomes. The certainty of the evidence was very low. We classified this indication in scenario nr. 5 (Table 1).

## Discussion

The dosimetry improvements of protons over photons are well-established [96]. However, the high costs of setting up PT facilities have somewhat limited their implementation and the possibility of conducting clinical research. Currently, there is still not enough evidence to confirm that dosimetry benefits have a significant clinical impact across all cancer types. In this context, the main objective of the HTA report carried out by AQuAS and published in 2024 [7] was to evaluate the clinical impact of PT, compared to XRT, in those cancer types, where the use of PT was not yet approved in Spain.

The main strength of the HTA report and of the present summary article lies in its foundation on a systematic review of published primary studies, conducted through a comprehensive systematic search across multiple health sciences databases and supplementary sources. The screening process was performed in parallel and independently by two evaluators following adapted Cochrane methodologies. Filters were applied based on publication date, including only studies published from 2012 onwards, to ensure relevance to current clinical practice. A modified GRADE approach was used to synthesize the results and assess the certainty of the evidence. The GRADE system is a widely adopted framework for evaluating the certainty of the evidence and the strength of healthcare recommendations. It was developed to address limitations of previous approaches and ensure a transparent, structured, and consistent process. The GRADE system considers multiple domains of evidence quality, direct link between evidence and recommendations, and promotes clarity in communication [97]. The inherent complexity of conducting randomized, unbiased, and precise comparative studies with PT meant that the strength of evidence based on GRADE was very low for almost all tumor types, preventing any recommendation for its use. ( 1), To address this, the evaluation team defined an evidence-availability scenario for each indication (Table 1) to assess whether PT could be considered for treatment using a calculated risk-taking approach..

Some limitations should also be acknowledged. First, language filters were applied during the study selection phase, restricting the inclusion to studies published in English, Spanish, and Catalan. Second, although the evaluation of the risk of bias was done as rigorously as possible following common rules among all evaluators, it might still be subject to a certain degree of subjectivity. Finally, during the GRADE assessment, inconsistency was deemed as “not applicable” when there was only one study per tumour type. Consequently, cancer types with limited available studies have been evaluated based on 3 instead of 4 domains, which might have potentially favoured these cases over those with a greater number of studies.

The results of the assessment report showed that there was no cancer type with enough evidence of enough certainty to be able to recommend or suggest the use of PT over XRT. However, we identified three cancer types, where the evidence seemed to indicate that PT was equivalent or superior compared to XRT in at least one primary safety outcome and one primary efficacy/effectiveness outcome; the evidence reached at least a low level of certainty for both a primary safety and a primary efficacy/effectiveness outcome. This scenario was observed for: (1) leptomeningeal metastases, where PT showed equal or superior results in terms of acute severe AEs, OS, and PFS compared to XRT, with low certainty of the evidence, but no published evidence was identified regarding chronic severe AEs; (2) lung cancer, where PT showed equal or superior results in terms of OS and G ≥ 3 radiation pneumonitis compared to XRT, with moderate and low certainty of the evidence, respectively, but with a very low certainty of the evidence regarding acute severe AEs and PFS; (3) anal cancer, where PT showed equal or superior results in terms of acute and chronic severe AEs and PFS compared to XRT, with low certainty of the evidence; however, no published evidence was identified regarding OS. It is worth noticing that the findings regarding cerebral leptomeningeal metastasis and anal cancer were based on only 1 study each; consequently, as mentioned in the limitations section, they might have been favoured over cases with a larger number of studies because no assessment of inconsistency was done. Therefore, the conclusion regarding these two tumor types is less robust than that of lung cancer.

In a third scenario, results were available for at least one primary safety outcome and one efficacy/effectiveness outcome. The findings suggested that PT may be an equivalent or superior strategy compared to XRT, but with a low or greater certainty of the evidence in only one of the domains (safety or efficacy/effectiveness). This scenario applied to glioblastoma, esophageal cancer, and prostate cancer. Five additional indications were identified, where the evidence tended to suggest that PT could be equivalent to or better than XRT in terms of safety and efficacy/effectiveness. However, the certainty of the evidence was very low, and therefore, the findings have to be interpreted with caution, carefully considering the source and limitations of the evidence in each case. The results may, however, help to identify indications, where favorable outcomes are more likely to be observed in the future. These included oral cavity and pharyngeal cancer, nasal cavity and paranasal sinus cancer, pancreatic cancer, hepatocellular carcinoma, and breast cancer. Finally, four other indications were identified, where either no primary outcomes were found (testicular cancer), or evidence was available for primary safety outcomes but not for effectiveness, or vice versa ( acoustic neuroma or vestibular schwannoma, uterine cancer, and medulloblastoma). As a result, it was not possible to assess the balance between potential benefits and risks of PT compared to XRT in these cases.

Demonstrating a significant benefit of PT over XRT has proved to be very complex. First, there is limited availability of PT equipment. Although in recent years, the number of available machines has increased significantly around the world [98], it is still very small compared to photon linacs. This translates into a lower capacity to treat patients and obtain clinical data. This challenge is even greater when it comes to performing clinical trials.

Second, in almost all comparative studies of PT vs XRT, the primary aim is to demonstrate a reduction in toxicity. This gives rise to specific methodological challenges that must be carefully addressed to ensure valid and clinically relevant results [99]. Although toxicity scales are used for the primary objective, toxicity is often a composite endpoint, and the assessment is more subjective. Estimating sample size may be difficult as well, and differences in safety may be more subtle than those related to clinical efficacy and effectiveness. Larger sample sizes are needed to detect differences with adequate statistical power with regard to toxicity outcomes [100]. Late toxicities, especially severe ones, require long-term follow-up for evaluation. For instance, more than 10 years might be needed to detect radiation-induced secondary cancers [101] or coronary heart disease [102]. This raises the question as to whether it is feasible to design RCT that require such a long follow-up period to demonstrate clinical benefit.

Third, comparative studies with PT vs XRT often have a high risk of bias. One of the key biases to highlight is confounding bias: outside the context of a RCT, patients who receive treatment with PT are often a selected group, in the sense that they have been chosen to receive this therapy. This selection is primarily based on the clinician’s decision—according to disease characteristics or technology availability—or on the patient’s own decision. At the same time, the patient’s decision is often influenced by factors, such as health insurance coverage and reimbursement policies, especially in contexts like the United States, where only certain patients have access to high-cost technologies such as PT. Although several identified CO studies applied strategies to mitigate the risk of bias, mainly through confounder-adjusted analyses and matching between groups using techniques such as propensity score matching, not all potential confounding variables may be available for adjustment, and it may not be possible to match all patients from one group with those of the other. As a result, these techniques rarely allow the level of evidence certainty to match that of an RCT, and the risk of bias may lead to an overestimation of the benefits and underestimation of the toxicity of PT.

Last but not least, another elusive aspect of interpreting trial results relates to generalizing the study findings to rapidly evolving clinical practice. A lot of research has been done analyzing results obtained using outdated technology (passive scattering) at a time when a more precise technology (pencil-beam scanning) is used. It is important to address the question about how the potential benefits of rapidly evolving technologies can be evaluated.

The HTA report on which this article is based generated a necessary debate in Spain and led to starting the work for the establishment of a national registry of patients treated with PT, similar to the ongoing European Particle Therapy Network (EPTN) [103] and pediatric registry initiatives [104]. This registry will promote the standardization and creation of protocols to collect treatment characteristics and outcomes, and will foster the generation of high-quality evidence on clinical outcomes in the medium and long term.

## Conclusions

PT faces challenges in proving significant clinical benefits over XRT. High facility costs and technological progress limit research, while the limited number of PT centers restricts patient access and the ability to conduct large clinical trials. Following an adapted GRADE methodology, we systematically assessed and synthesized the available evidence regarding the safety and efficacy and effectiveness of PT compared to XRT. While no cancer type met the highest level of evidence according to GRADE, the evidence suggested potential benefits of PT in three indications (lung cancer, anal cancer, and leptomeningeal metastases). In these cases, PT showed equal or improved outcomes in toxicity and no differences in terms of survival; however, the certainty of the evidence was low. Additional indications (glioblastoma, esophageal cancer and prostate cancer) showed promise but only showed some improvement in either the efficacy or the safety domains, not both. These results support investigating PT in specific clinical settings, where the benefits of personalized treatment may outweigh the current limitations of the evidence.

## Data Availability

The data used for the preparation of this article are included in the assessment report published by AQuAS and available on the following link: http://hdl.handle.net/11351/12999
